# Radiotherapy and COVID-19—everything under control or just the start of a long story?

**DOI:** 10.1007/s00066-020-01704-x

**Published:** 2020-11-24

**Authors:** Ursula Nestle, Mechthild Krause

**Affiliations:** 1 Klinik für Strahlentherapie und Radioonkologie, Kliniken Mariahilf Mönchengladbach, Mönchengladbach, Germany; 2 Klinik für Strahlenheilkunde, Universitätsklinikum Freiburg, Freiburg, Germany; 3 German Cancer Consortium (DKTK) partner site Freiburg and German Cancer Research Center (DKFZ), Heidelberg, Germany; 4 German Cancer Consortium (DKTK) partner site Dresden and German Cancer Research Center (DKFZ), Heidelberg, Germany; 5 OncoRay-National Center for Radiation Research in Oncology: Faculty of Medicine and University Hospital Carl Gustav Carus, Technische Universität Dresden, Helmholtz-Zentrum Dresden-Rossendorf, Dresden, Germany; 6 Dept. of Radiation Oncology, Faculty of Medicine and University Hospital Carl Gustav Carus, Technische Universität Dresden, Dresden, Germany; 7 Institute of Radiooncology-OncoRay, Helmholtz-Zentrum Dresden-Rossendorf, Dresden, Germany; 8 National Center for Tumor Diseases (NCT), Partner Site Dresden, Germany: German Cancer Research Center (DKFZ), Faculty of Medicine, University Hospital C.G. Carus Dresden and Helmholtz-Zentrum Dresden-Rossendorf, Dresden, Germany

This pandemic is an imposition! We all are exhausted by repeated discussions on the current situation. Would like to be “back to normal”—whatever that might be—very soon.

Unfortunately, this is still a dream and we are in the middle of the corona reality. Almost forgotten: the initial panic that radiooncology could no longer operate according to law under pandemic conditions was quickly and effectively countered by an unprecedented concerted response from the authorities.

Then we had all these practical questions: How to deal with potentially limited personnel resources? How to treat potentially infected patients in routine care? To this end, at a very early stage, the German Society for Radiooncology (DEGRO), together with the Working Group for Radiooncology (ARO) of the German Cancer Society and the National Association of German Radiotherapists (BVDST), compiled two helpful statements and recommendations [1–3].

At a previously unimagined speed, we then dealt with hygiene concepts, made friends with hypofractionation, optimized our workflow, discussed home office solutions, formed staff groups, and reorganized the aftercare outpatient clinics. In their interesting survey in this issue, Matuschek et al. report on how well all this has worked out [4]. Overall, in Germany, only a relatively small number of COVID-positive patients have had to be treated by radiotherapy so far. We will see how the situation will develop during the remainder of this year. At least we are very well prepared—both with concepts and organizational skills—for higher infection rates.

Three aspects may change our working life beyond the pandemic:

- Changing workflows, home office capabilities, digital collaboration. This is what the group from Bellinzona [5] vividly describes—a model for the future?
- Hypofractionation, which “before corona” entered our German routine only slowly for many tumor entities, will certainly retain greater importance. As already stated in the DEGRO statement, it is crucial that herefor published dose/fractionation schedules from randomized phase III studies are used and that in every institution the introduction of new concepts is closely monitored clinically and organizationally. A “living” overview of published hypofractionated radiation regimens for many indications (including the corresponding normal tissue constraints) is available at: https://docs.google.com/spreadsheets/d/1KicEMU_ZZ5rcpCEmNDelQcDOdYqZ4iMzh64bx36ac58
- Our methods for radiooncological aftercare may also change: DEGRO’s respective statement from back in 2015 already considers telephone interviews [6]. Now, thanks to the current boost of digitalization, online consulting is also increasingly possible. However, despite all the useful rationalization, we must not forget the importance of structured personal aftercare, especially for patients who are mentally stressed or those with communication problems due to their illness.

Early on in the pandemic another issue arose, which may deserve our attention: The idea of irradiating the lungs of patients with COVID-19 pneumonia with very low doses in order to achieve an anti-inflammatory effect, in analogy with the “traditional” radiotherapy of benign diseases. Described in a publication by Calabrese and Dhawan from 2013 [7], which summarizes experiences with irradiation of viral and bacterial pneumonia from the first half of the 20th century, this concept was taken up very early in corona-plagued Iran [8] and also discussed, e.g., in Canada. Two case reports on this topic can be found in this volume of *Strahlentherapie and Onkologie* [9, 10]. In their review, Rödel et al. conclude that biologically, there is indeed hope for reproducible anti-inflammatory effects [11]. Preclinical and clinical studies are ongoing internationally, 16 clinical studies on low-dose irradiation and COVID-19 are already registered in the register platform clinicaltrials.gov and the topic is also being discussed in Germany. A success of these studies might bring a renaissance of low-dose radiation therapy for benign diseases—already declared dead. However, beyond a not yet prospectively proven effect on the course of viral pneumonia, some important aspects must be kept in mind during the evaluation and discussion of this idea:

- Is an inhibition of the pulmonary inflammatory response in COVID patients really relevant for prognosis or are the vascular complications more likely to be decisive for the outcome?
- Are there alternative, e.g., drug-based, therapeutic approaches that can be realized with less effort, at least in health care systems like ours, for patients requiring intensive care?
- What about the increasing lifetime risks for malignancies (0.6–4 excess lung cancer/100 patients) and cardiovascular deaths (0.8–7.6 extra death/100 patients), if we burden the radiation-sensitive thoracic organs of younger “benign” patients with 0.3–1 Gy? [12].
- How can oncological patients and radiotherapy staff be optimally protected from infection and competition for equipment and personnel resources in the event of planned radiation, if many severely ill COVID cases should receive radiotherapy?

As least: radiooncology is subject to speedy developments, even in times of pandemics! In our opinion, the most important thing is the continuous contribution to the optimal care of cancer patients, with and without COVID-19. A certain diagnostic gap—due to the lack of preventive examinations and reduced patient willingness to visit a doctor—might already be disadvantageous enough.

Potentially confronted with increasing numbers of COVID-19 infections in the coming autumn and winter, our solidarity will be necessary: before shortages of resources caused by the pandemic may cause unplanned breaks, treatment discontinuations, or long delays in radiotherapy onset, we must activate our existing neighborhood cooperation agreements and therefore test the resilience of our professional network. It would be an excellent signal if radiooncology as a whole could guarantee the treatment of cancer patients across institutions at guideline level!

Yes: this pandemic is an imposition! We really hope for a mild end, not too long from now—but perhaps the current crisis may still leave some positive traces in radiooncology.
